# Male Obesity Associated Gonadal Dysfunction and the Role of Bariatric Surgery

**DOI:** 10.3389/fendo.2020.00408

**Published:** 2020-06-19

**Authors:** Sana Sultan, Ameet G. Patel, Shamsi El-Hassani, Benjamin Whitelaw, Bianca M. Leca, Royce P. Vincent, Carel W. le Roux, Francesco Rubino, Simon J. B. Aywlin, Georgios K. Dimitriadis

**Affiliations:** ^1^Department of Endocrinology and Metabolic Medicine, King's College Hospital NHS Foundation Trust, London, United Kingdom; ^2^Department of Minimal Access Surgery, King's College Hospital NHS Foundation Trust, London, United Kingdom; ^3^Minimal Access and Bariatric Unit, Princess Royal University Hospital, King's College Hospital NHS Foundation Trust, Orpington, United Kingdom; ^4^Department of Clinical Biochemistry, King's College Hospital NHS Foundation Trust, London, United Kingdom; ^5^Diabetes Complication Research Centre, School of Medicine and Medical Science, UCD Conway Institute, University College Dublin, Belfield, Ireland

**Keywords:** obesity, male reproductive system, male obesity related hypogonadism, bariatric surgery, metabolic surgery, male subfertility, oligospermia, erectile dysfunction

## Abstract

Obesity is an ever growing pandemic and a prevalent problem among men of reproductive age that can both cause and exacerbate male-factor infertility by means of endocrine abnormalities, associated comorbidities, and direct effects on the precision and throughput of spermatogenesis. Robust epidemiologic, clinical, genetic, epigenetic, and preclinical data support these findings. Clinical studies on the impact of medically induced weight loss on serum testosterone concentrations and spermatogenesis is promising but may show differential and unsustainable results. In contrast, literature has demonstrated that weight loss after bariatric surgery is correlated with an increase in serum testosterone concentrations that is superior than that obtained with only lifestyle modifications, supporting a further metabolic benefit from surgery that may be specific to the male reproductive system. The data on sperm and semen parameters is controversial to date. Emerging evidence in the burgeoning field of genetics and epigenetics has demonstrated that paternal obesity can affect offspring metabolic and reproductive phenotypes by means of epigenetic reprogramming of spermatogonial stem cells. Understanding the impact of this reprogramming is critical to a comprehensive view of the impact of obesity on subsequent generations. Furthermore, conveying the potential impact of these lifestyle changes on future progeny can serve as a powerful tool for obese men to modify their behavior. Healthcare professionals treating male infertility and obesity need to adapt their practice to assimilate these new findings to better counsel men about the importance of paternal preconception health and the impact of novel non-medical therapeutic interventions. Herein, we summarize the pathophysiology of obesity on the male reproductive system and emerging evidence regarding the potential role of bariatric surgery as treatment of male obesity-associated gonadal dysfunction.

## Introduction

Obesity is a multifactorial global pandemic, which affects virtually every system in the body and can have significant bio-psycho-social impacts on the patients. It is increasingly becoming a significant health issue, which now affects all age groups and is seen in the developed and developing world (1). In the year 2016, the World Health Organization (WHO) data showed that 39% of the world's population is overweight and 13% are obese (1).

Obesity results from complex interactions between metabolic, genetic, environmental, nutritional, and psychosocial factors. Excess body weight has been established as a clear risk for increased morbidity and mortality and is a known risk factor for several serious physical health conditions such as hypertension, type II diabetes mellitus (T2DM), dyslipidemia, cerebrovascular, and cardiovascular illness (2). In addition we now know that obesity also impacts both male and female sexual and reproductive function (2). Throughout history and pervading many civilizations the penis in an erect state has been a powerful symbol of male fertility, virility, and dominance. Therefore, the inability to achieve and/or maintain an erection can have profound negative physical and psychological effects on the patient as an individual and indirectly on the partner (3).

## Aetiopathogenesis of Male Obesity Related Hypogonadism

### Testosterone Deficiency

The male reproductive system is a multilevel complex physiological system that is controlled by various feedback mechanisms and signaling molecules, which are all in a delicate balance. Hypogonadism is defined as decreased or absent gonadal function and in males is characterized by reduced testosterone levels (4). There exists an expectation that testosterone levels will naturally decline with age and can lead to or strongly contribute to hypogonadism (5). Obesity and in particular, visceral adipose tissue expansion, represents a strong risk factor for the development of male hypogonadism and this state is referred to as male obesity secondary hypogonadism (MOSH) (6). The effects of MOSH will manifest themselves as testosterone deficiency, erectile dysfunction, changes in sperm, and semen parameters and overall sexual dysfunction (6). The current scientific literature shows established links between obesity and secondary hypogonadism where one cross sectional study has demonstrated an 8.7-fold increase in risk of secondary hypogonadism in patients with a BMI >30 kg/m^2^ (7).

Furthermore, men with a BMI of 35–40 kg/m^2^ can have up to 50% less free and total testosterone when compared to age matched peers with a normal BMI (8). There is overwhelming undisputed evidence of the link between obesity and male sexual and reproductive dysfunction however the exact mechanisms for this remains to be elucidated (7–9).

**Figure 1:**
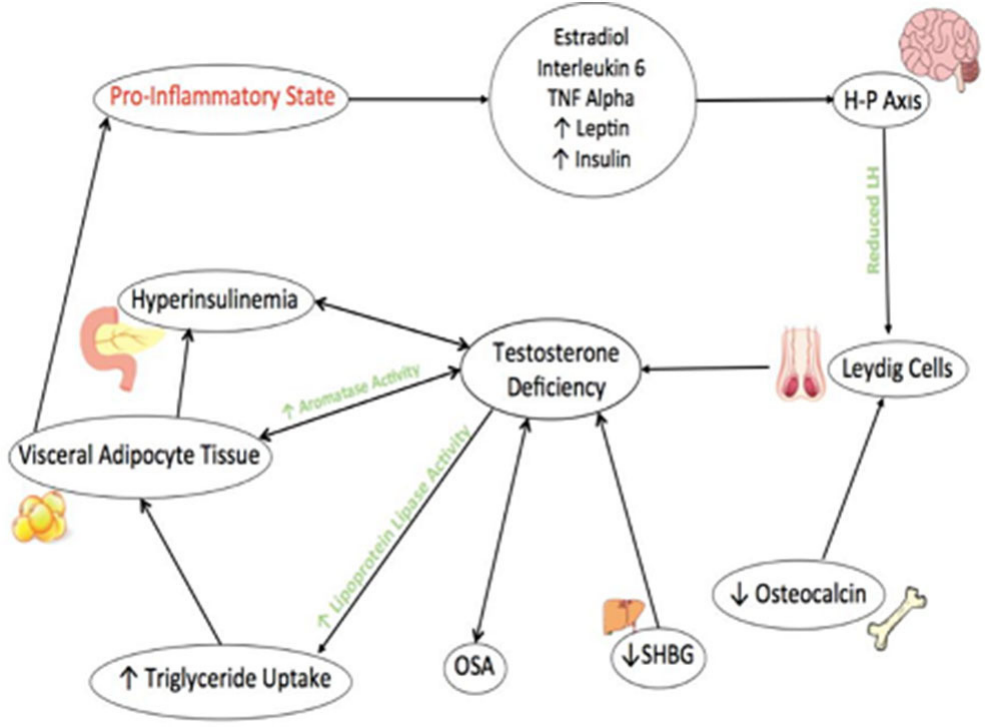
The metabolic and reproductive effects of testosterone in relation to obesity. Factors related to obesity, such as OSA and reduced SHGB contribute to Testosterone deficiency. Reduced Osteocalcin output from Osteoblasts is also newly associated with contributing to testosterone deficiency. Testosterone deficiency increases activity of Lipoprotein Lipase and therefore triglyceride uptake which increases visceral adipose tissues. This further exacerbates htperinsulinemia and testosterone deficiency through the activities of Aromatase enzyme. Visceral adipose tissue increases production of cytokines and inflammatory mediators leading to a pro-inflammatory and contribute to hypothalamic-pituitary suppression leading to further testosterone deficiency.

The Cohen hypothesis was the first suggested link between testosterone and obesity in the literature (10). This is based on the observation that testosterone and obesity have a circular relationship in which obesity acts as a strong independent risk factor to testosterone deficiency (10). Similarly suboptimal testosterone levels can in their own right exacerbate obesity leading to a vicious cycle, which can only be broken with substantial reduction in body mass (10). Figure 1 shows this relationship and highlights other contributing causes.

Testosterone is known to be a hormone that has potent metabolic effects and it is now accepted that testosterone levels and subsequent effects are closely related to body mass although the exact mechanism remain to be elucidated (11). In suboptimal levels, testosterone will facilitate the differentiation of pluripotent stem cells into adipocytes, which will cause increased aromatization of testosterone into oestradiol (11). This will act via negative feedback mechanism to the hypothalamic –pituitary axis (HPA) to suppress further gonadal stimulation and testosterone release (11).

In addition to those mechanisms in Cohen's hypothesis, testosterone deficiency is known to play indirect metabolic roles preventing physiological compensatory mechanisms (11). This is explored in the hypogonadal—obesity—adipocytokine hypothesis, which expands this idea further (11). Testosterone is known to enhance the activity of lipoprotein lipase enzyme, which leads to a rise in triglyceride uptake into adipocytes (11). Furthermore, larger numbers of adipocytes lead to increases in insulin resistance, production of pro-inflammatory cytokines tumor necrosis factor-a. interleukin-1 and interleukin-6 (TNF-α, IL-1, and IL-6), and increases in leptin and oestradiol levels (11, 12).

Leptin is a hormone produced by adipocytes, and acts to regulate energy balance ensuring reduced fat storage (13). It also has other roles and acts at the hypothalamic- pituitary level to influence the reproductive system in both male and female patients (13). In normal conditions, Leptin potentiates the release of testosterone, by acting at the hypothalamic level, stimulating neurons to release gonadotrophin releasing hormone (GnRH), and subsequently luteinizing hormone (LH) from the pituitary gland (13). However, in obesity these neurons become resistant to the actions of Leptin (11, 13). In addition, receptors for Leptin have been found in testicular tissue and studies have shown that Leptin can directly inhibit gonadotrophic actions on Leydig cells thus further exacerbating testosterone deficiency and therefore obesity (11, 13).

The initial signals that activate the hypothalamic- pituitary-gonadal axis originate in the arcuate nucleus and periventricular nucleus of the hypothalamus (14). These neurons release neuropeptide Kisspeptin, which in turn stimulates the release of GnRH (14). Excess levels of oestradiol and pro-inflammatory cytokines act at the hypothalamic level to inhibit the neuronal release of Kisspeptin, which will lead to a profound reduction in GnRH signals leading to a sustained state of hypogonadotropic hypogonadism (12, 15).

Another hypothesis for male hypogonadism in obesity is the Gelding theory. Currently, within the scientific literature there has been more evidence supporting this theory as at least contributing to testosterone deficiency. Within the gastrointestinal system there exists on average 1.5 kg (100 trillion) of bacteria (16, 17). This tends to be largely gram-negative bacteria, which produce a pro-inflammatory lipopolysaccharide and endotoxin (16, 17). Under normal circumstances these proinflammatory substances are confined to the gastrointestinal tract (16, 17). However, a highly calorific and fatty diet has been shown to contribute to breakdown in the intestinal mucosa leading to passage of bacterial endotoxins into the general circulation, which further contributes to a pro-inflammatory state (16, 17). Exposure to lipopolysaccharide from the general circulation is hypothesized to cause impaired testicular function thereby contributing to testosterone deficiency. The exact mechanism of how this occurs remains unclear (16, 17).

Sex Hormone Binding Globulin (SHBG) is a glycoprotein produced by the liver. In the circulation, ~98% of testosterone is bound to albumin and SHBG (18, 19). Suboptimal SHBG levels can exacerbate testosterone deficiency through pro-inflammatory effects and increased deposition of lipids within macrophages and adipocytes. This will exacerbate the existing inflammatory state of obesity and propagate further weight gain (18). In addition, it is now accepted that suboptimal levels of SHBG and testosterone significantly contribute to insulin resistance and therefore a pre-diabetic state. The MRFIT and the Massachusetts Male Aging Study followed up 176 and 1,156 patients over 5 and 7–10 years, respectively (20, 21). Both showed results that are consistent with the wider literature, which is that low levels of testosterone and SHBG were strongly associated with the development of diabetes (20, 21). In obese patients there exists a state of hyperinsulinemia due to reduced insulin sensitivity in peripheral tissues (6). This leads to high levels of insulin, which have been shown to contribute to firstly central hypogonadism through the modulation of GnRH and therefore gonadotrophin output and secondly to peripheral hypogonadism through direct actions on Leydig cells (6, 22, 23).

Testosterone levels are also influenced by Obstructive Sleep Apnea (OSA), which is a common condition and often co-exists with obesity and excess body weight (24, 25). The underlying pathophysiological mechanism of OSA involves partial or complete upper airway obstruction, which results in intermittent hypoxia, sleep fragmentation, reduced deep, and rapid eye movement (REM) sleep and daytime somnolence (24, 25) OSA has been associated with pituitary-gonadal dysfunction in male patients, which is manifested as suboptimal testosterone levels, erectile dysfunction, and reduced libido (24, 25). Suboptimal testosterone levels found in OSA are due to reduced quality and quantity of sleep, which in turn worsens both obesity and OSA (24, 25).

### Erectile Dysfunction

Testosterone deficiency also leads to difficulties in achieving and maintaining an erection. Healthy erectile function is dependent on interactions of erectile tissue, central, and peripheral nervous system, several endocrine, and psychological factors (3). Sexual stimulation results in activation of non-adrenergic, non-cholinergic, and parasympathetic cholinergic nerve fibers leading to the release of nitric oxide and acetylcholine, respectively (3). This will lead to increased cyclic guanosine monophosphate (GMP) and reduced intracellular calcium levels thus facilitating the relaxation of smooth muscles and vasodilatation causing increased blood flow to corpus cavernosum causing veno-occlusion of the subtunical venules (3). Disturbances in one or more of these mechanisms can contribute or lead to erectile dysfunction (3, 26).

Excess body weight has been strongly linked to erectile dysfunction and in fact current research has suggested high BMI as a significant risk factor for erectile dysfunction (27, 28). Obesity leads to a pro-inflammatory physiological state, which favors widespread arteriolar endothelial dysfunction and impaired activity of nitric oxide. In addition, it also leads to increased insulin and leptin resistance, which can exacerbate T2DM and resultant peripheral neuropathies (29). Obesity is also known to cause both central and peripheral hypogonadism and testosterone deficiency thus further exacerbating erectile dysfunction (29).

Current literature has shown that patients with a raised BMI have a 30% increased risk of erectile dysfunction than those of a normal BMI, and 96.5% of obese patients with metabolic syndrome report erectile dysfunction (30, 31). A multivariate analysis conducted by Bacon et al. showed an increased risk of erectile dysfunction at 19 and 33% in men with a BMI of 25–26.9 and 27–29.9kg/m^2^, respectively (32, 33). The risk of erectile dysfunction is further compounded in patients with obesity due to the presence of other co-morbid conditions such as cardiovascular dysfunction and T2DM (29, 34). A meta-analysis investigating the prevalence of erectile dysfunction in T2DM showed that 52.5% of diabetic patients report erectile dysfunction giving prevalence odds of approximately 3.5 times more than healthy individuals (34).

Erectile dysfunction is essentially reflective of underlying vascular disease in those patients with obesity and some researchers have suggested that the presence of erectile dysfunction can be used as a prompt for further assessment of cardiovascular system (28).

### Sperm and Semen Parameters

Obesity has a clear negative effect on male reproductive and sexual function, however the effect of obesity on sperm and semen parameters remains ambiguous. It is wholly accepted that obesity increases oxidative stress through a prolonged and sustained pro-inflammatory state, which is hypothesized to cause oxidative damage to the sperm. This is thought to lead to decreased motility, increased DNA damage, reduced acrosomal reactions, and therefore lower successful outcomes following *in vitro* Fertilization (IVF) (6, 35).

Some studies have suggested that elevated BMI results in a detrimental effect on sperm morphology, motility, and concentration however others have found no association (36–38). Belloc et al. investigated 10,665 semen samples and found that in morbidly obese patients (BMI >40 kg/m^2^) there was reduced semen volume, concentration, total sperm count, and reduced motility (39). There were also significant increases found in azoospermia and cryptozoospermia from 1.9 and 4.7% to 9.1 and 15.2%, respectively (39). Similarly Sermondade et al. carried out a systemic review investigating the effect of BMI on sperm count. They found that compared to men of normal BMI, those in the obese or morbidly obese categories had an odds ratio of 1.28 (1.06–1.55) and 2.04 (1.59–2.62) for oligozospermia and azoospermia, respectively (40). In contrast, a cross sectional study by Rufus et al. carried out a comparison of semen quality in 206 men of varying BMI (41). The overall outcome of the study was that elevated BMI did not have a significant effect on semen quality (41).

It is clear that there is no consensus in the current literature regarding the effects of obesity and excess body weight on sperm and semen parameters. Further research is required into this area so that we may better understand the effects of obesity on male reproduction. This will inevitably allow the provision of more targeted and therefore more effective treatment for reproductive issues in male patients with obesity.

### Effects of Paternal Obesity Through Future Generations

Intragenerational inheritance occurs when induced epigenetic changes in the gametes are passed on to the subsequent generation (42). This epigenetic inheritance can lead to changes to the phenotype of the offspring and beyond generations. Transmission of epigenetic signatures for the paternal gamete, includes any sperm epigenetic signatures altered throughout a man's life (or even that were induced *in utero*) from the father to the child only and termed intergenerational. Whereas, the inheritance of an altered epigenetic state beyond immediate offspring is termed transgenerational inheritance. According to epidemiological evidence, many types of environmental challenges imposed on the parent, such as hunger, specific diets, toxins, and trauma, have been found to influence the development of the offspring (43). Paternal age, subfertility, smoking, obesity, and exposure to a range of environmental influences, including air pollution, radiofrequency electromagnetic radiation and others, have also been implicated (44). The Dutch famine study showed that severe caloric restriction in parents during wartime starvation in World War II, resulted in significant cardiometabolic abnormalities in the *post-utero* life of their offsprings (45).

Obesity has been shown to directly drive transgenerational effects in successive generations. Preclinical studies with the use of animal models have found altered metabolic processing in offspring sired by obese fathers (46). Further studies using murine models have shown similar patterns of offspring metabolic perturbations but have also identified specific epigenetic alterations in the sperm (47). In humans, an intriguing recent study found alterations in DNA methylation at the insulin-like growth factor 2 gene in newborns sired by obese fathers (48). Taken together, data from experimental studies strongly suggest that obesity can affect sperm function, embryogenesis, and even offspring health; though, the same results have not been clearly demonstrated in humans (49, 50).

Donkin et al., demonstrated that the expression level of specific miRNAs, and small nuclear RNA (snRNA) fragments was altered in the spermatozoa of obese men. They speculated that this altered expression coordinately modulated the expression of genes involved in behavior and food intake and could participate in predisposing the offspring to obesity (50).

## Management of Male Obesity Related Hypogonadism

### Non-surgical Interventions

Current management of obesity entails access to specialized secondary and tertiary care services that allow patients to engage with a multidisciplinary team to aid weight loss and maintenance. Lifestyle modifications such as caloric deficit diets and increased physical activity are the mainstay of non-surgical therapies. Recently, treatment with a Glucagon Like Peptide−1 (GLP-1) receptor agonist has shown beneficial effects with regards to weight loss but have also shown increased levels of gonadotrophins and subsequent increases in testosterone (50).

Several studies have demonstrated benefits with regards to improvement and recovery in the male reproductive system following significant and sustained weight loss. The European Male Aging Study (EMAS) has shown that changes in total body weight of ≥15% are correlated to changes in testosterone whereby gaining weight reduces testosterone levels and vice versa (51). Similarly the Massachusetts Male Aging Study established that a 4–5 kg/m^2^ increase in BMI significantly contributes to a reduction in total testosterone level comparable to 10 years of aging (52). Hakonsen et al. also demonstrated similar results in a pilot cohort study of 43 men who were enrolled in a residential weight loss program. This study showed an average weight loss of 15%, which correlated to improvement in hormonal profile and an increase in total sperm count and semen volume (53). Mir et al. also showed that weight loss leads to significant reduction in DNA fragmentation index and therefore improved sperm morphology (37).

In a cross-sectional prospective cohort study, Bacon et al. demonstrate that physical activity is associated with 30% decreased risk of erectile dysfunction (31). Further studies have explored the relationship of weight to erectile function and have used the International Index of Erectile Function (IIEF) score. One such study demonstrated that lifestyle measures can cause a reduction in BMI from 36.9 to 31.2 kg/m^2^ and improvement in IIEF score from 13.9 to 17, respectively (54).

### Bariatric Surgery: Hormonal and Metabolic Effects

Intense and sustained lifestyle modifications can show adequate weight loss of up to 10% at 1 year with up to 5.3% maintained at 8 years (55). However, a subset of obese and morbidly obese patients will require surgical interventions in the form of bariatric surgery (55). Eligibility for surgical intervention in the treatment of obesity is globally variable; however, it is generally accepted that bariatric surgery could be an appropriate option for patients with a BMI >40 kg/m^2^ or those with a BMI of 35–40 kg/m^2^ with co-morbid conditions (55). There are different surgeries available however all seem to show favorable outcomes. In a systematic review by O'Brien et al., patients achieved 71, 60, and 49% excess weight loss with a biliopancreatic diversion with duodenal switch (BPD/DS), laparoscopic Roux en Y gastric bypass (RYGB) and laparoscopic adjustable gastric band (LAGB), respectively. (56, 57). Such drastic and sustained weight loss will have profound effects on alleviation of obesity and associated co-morbid conditions. Table 1 shows relevant studies investigating the effect of bariatric surgery on hormonal and metabolic profile, erectile dysfunction, and semen and sperm parameters.

**Table 1:** Effects of bariatric surgery on hormonal and metabolic profile, erectile dysfunction, semen and sperm parameters.

**Level of** **evidence**	**References**	**Sample Size**	**Intervention**	**Outcome**
Level Ib	Samavat et al. (58)	103	Gastric bypass	Post operative time point review: 9 months Weight loss achieved: Mean −36.2 ± 20.24 kg Outcome: significant increase in free and total testosterone, OCN and SHBG; decrease in Oestradiol
Level Ib	Arolfo et al. (59)	44	Roux-en-y gastric bypass; Sleeve gastrectomy	Post operative time point review: 9 months Weight loss achieved: Mean-39.75 ± 24 kg Outcome: Significant increase in total testosterone, SHBG and IIEF score; decrease in HbA1c, insulin, triglycerides, HDL cholesterol, and CRP
Level Ib	Legro et al. (60)	6	Roux-en-y gastric bypass	Post operative time point review: 1, 3, 6 months Weight loss achieved: Mean −55 ± 30 kg Outcome: improvement in testosterone level
Level Ib	Reis et al. (61)	20	Lifestyle modifications, Gastric bypass	Post operative time point review: 4 and 24 months Weight loss achieved: inaccessible Outcome: improvement in total and free testosterone and FSH and reduced prolactin levels
Level IIb	Liu et al. (62)	45	Roux-en-y gastric bypass	Post operative time point review: Weight loss achieved: inaccessible Outcome: increase in total testosterone with reduced visceral fat
Level IIIb	Calderon et al. (63)	35	Gastric bypass; Sleeve gastrectomy; Gastric Banding	Post operative time point review: 6 months or more (not specified) Weight loss achieved: −51.0 ± 20.7 kg for RYGB group and −31.1 ± 17.6 kg for the restrictive surgery group Outcome: increase in total testosterone and SHBG; decrease in fasting glucose and insulin
Level Ia	Glina et al. (64)	7 articles	Bariatric surgery—Review article	Outcome: improvement in IIEF score
Level IIb	Dallal et al. (65)	97	Gastric bypass	Post—operative time point review: 19 months Weight loss achieved: Mean 53 ± 29 kg Outcome: improvement in sexual drive, erectile function, ejaculatory function, and sexual satisfaction
Level IIb	Kun et al. (66)	39	Roux-en-y gastric bypass	Post operative time point review: 12 months Weight loss achieved: Unspecified Mean pre-op BMI: 41.2 ± 8.5 kg/m^2^ and post-op 32.1 ± 7.3 kg/m^2^ Outcome: improvement in cavernosal thickness and vasculopathy, endothelial function scores, and IIEF
Level Ia	Wei et al. (67)	6 articles	Gastric bypass—Review article	Outcome: increase in semen volume; no changes in semen concentration or motility
Level IIa	Carette et al. (68)	46	Gastric bypass; Sleeve gastrectomy	Post operative time point review: 6 and 12 months Weight loss achieved: inaccessible Outcome: increase sperm count likely due to resolution of hypogonadism and DNA fragmentation
Level IIb	Samavat et al. (69)	31	Roux-en-y gastric bypass; Medical management	Post operative time point review: 0 and 6 months Weight loss achieved: Mean −34.7 ± 16.7 kg Outcome: improvement in total sperm motility and number; Statistically significant increase in semen volume. Reduced Il- 8 levels and sperm DNA fragmentation
Level IV	Sermondade et al. (70)	3	Roux-en-y gastric bypass; Sleeve gastrectomy	Post operative time point review: not specified Weight loss achieved: Unspecified Case 1: −32.3 kg/m^2^ Case 2: −23.1 kg/m^2^ Case 3: −11.1 kg/m^2^ Outcome: severe worsening of parameters in 2 patients; reversibility seen in 1 patient 2 years post-operatively

Levels of evidence based on Oxford Center for Evidence Based Medicine criteria (71).

*OCN, Osteocalcin; SHBG, Serum Hormone Binding Globulin; IIEF, International Index of Erectile Function; HDL, High Density Lipoproteins; CRP, C-Reactive Protein; FSH, Follicle Stimulating Hormone*.

In addition to significant and sustained weight loss, several studies have also demonstrated improvement in total testosterone, SHBG levels, insulin resistance, and several other metabolic parameters (63, 72). Interestingly, the improvements in these parameters are not procedure specific (63, 72). Extensive and sustained weight loss leading to improvement in MOSH has consistently been reported in the literature. In a systematic review and meta-analysis of 45 articles, MOSH was present in 64% of patients preoperatively with resolution seen in 87% of patients post-operatively (73). Similarly, Pellitero et al. carried out an observational study of 33 men undergoing bariatric surgery and showed that 1 year post-operatively there was significant excess weight loss observed at 18.8% with an increase in total and free testosterone, SHBG and FSH and decrease in estradiol and prolactin (63).

Further studies have also demonstrated that decreases in visceral adipose tissue leads to increases in free testosterone and these effects are thought to be long lasting (6, 74). Visceral adipose tissue is known to contain higher levels of aromatase enzyme thereby contributing to excess conversion of testosterone to estradiol (6, 74). Therefore, it would make sense that reduction in visceral adipose tissue could disturb this mechanism and lead to a rise in testosterone levels and therefore overall improvement in MOSH (6).

It is now accepted that obesity and excess body weight creates a pro-inflammatory physiological state that directly causes or exacerbates several health conditions including MOSH. Studies have shown favorable outcomes in terms of reduction in pro-inflammatory mediators following bariatric surgery. This is seen in a study of 20 morbidly obese subjects undergoing adjustable gastric banding. One year post operatively they had significant reduction in BMI from 41.6 to 30.8 kg/m^2^ and high sensitivity C Reactive Protein (hs-CRP) from 1.33 to 0.4 mg/dl, respectively (62). These results were replicated in a study of 47 patients (BMI >40 kg/m^2^), and post operatively, subjects were found to have lost an average of 33% of their body weight, which was correlated with a reduction of hs-CRP from 0.83 to 0.18 mg/dl (56, 75).

Bariatric surgery clearly shows immediate and sustained reduction in BMI and therefore improvement in pathophysiological factors causing or contributing to hypogonadism. Pham et al. have also shown that these effects do persist long term (71). They carried out a randomized controlled trial of 32 male obese patients assigned to medical therapy or surgical treatment (76). Surgical patients showed an increase of 47.4% in free testosterone compared to medical patients at a 2.2% increase only. The increases in testosterone also correlated to decreased high sensitivity CRP, leptin, and overall BMI (76).

Experimental evidence has suggested potential links between the skeletal and reproductive system. Osteoblasts produce a hormone called Osteocalcin, which acts directly on the Leydig cells in the testes to produce testosterone (77). Samavat et al. have shown that reduction in BMI leads to increased levels in Osteocalcin, which also correlates to an increased level of testosterone (78). It is widely accepted that testosterone is a hormone, which exerts significant metabolic effects in both direct and indirect manners (78). This is clearly an area that is promising however requires further research to elucidate exact effects and how dependent they may be on other risk factors such as age or other comorbidities.

### Bariatric Surgery: Erectile Dysfunction

There is agreement in the literature regarding the detrimental effects of obesity and excess body weight on erectile function. The impact of erectile dysfunction on the individual patient but also in a partnership is profound and can lead to significant negative effects on quality of life. This effect is ameliorated with reduction in BMI and improvements can be seen in biological and psychological factors contributing to reproductive and sexual function (58, 65). In a systematic review and meta-analysis assessing erectile function in patients with obesity pre- and post-operatively showed a 4.10 increase in the IIEF score demonstrating statistical significance (79). Furthermore, a study of 97 patients with obesity undergoing bariatric surgery showed improvement in sexual drive, erectile and ejaculatory function, and sexual satisfaction post operatively. These effects were directly correlated to percentage of excess body weight lost (64). In a prospective study by Arolfo et al. data was collected pre- and 12 months post-operatively on 44 male patients. Patients had significant improvement in IIEF 12 months after surgery (59).

Improvement in sexual function and specifically erectile function is clearly seen with bariatric surgery and correlated to improvements in hormonal profile. However, the positive effects seen on erectile function are thought to be due to more than just an improvement in hormonal and metabolic profiles. We know that erectile dysfunction in part results from issues with the integrity of blood vessels in penis. In fact, some researchers have also suggested that erectile dysfunction could be a manifestation of vascular disease and endothelial dysfunction (59). This theory was reinforced in a retrospective cohort study by Kun et al. in which 39 obese men were investigated and 46.2 and 53.8% had cavernosal vasculopathy and carotid vasculopathy, respectively (76). This reduced post bariatric surgery to 15.4 and 23.1% and subsequently led to an improvement in IIEF from 17.3 to 23.8 (80).

In reality, improvement in erectile function and in fact reversal of erectile dysfunction is likely due to a drastic and maintained reduction in excess weight leading to a more favorable hormonal and metabolic profile. Further amelioration of other co-morbidities and risk factors such as endothelial dysfunction clearly significantly contributes to better outcomes.

### Bariatric Surgery: Sperm and Semen Parameters

The effects of bariatric surgery on hormonal profiles and erectile function in male patients with obesity are clearly favorable. However, the impact on semen and sperm parameters remains inconsistent within the current literature. Samavat et al. assessed morbidly obese patients 6 months after laparoscopic RYGB and found improvement in overall semen volume and viability (66). In addition, they found a decrease in serum Il-8 levels and sperm DNA fragmentation (66).

Similarly, a systematic review and meta-analysis of 6 articles showed that patients who had undergone gastric bypass surgery had an increase in semen volume (69).

Conversely, Lee et al. carried out a systematic review and meta-analysis of 28 cohort studies in which sperm parameters were reported in only three of those articles and showed no statistically significant difference pre- and post- bariatric surgery (67). Furthermore the BARIASPERM study, assessing 46 male patients with no prior history of infertility, showed that sperm count at 6 and 12 months post operatively was significantly reduced (81). Patients underwent either a gastric bypass surgery or a sleeve gastrectomy with both showing reduced total sperm count at −41.1 million and −91.1 million, respectively (81).

The reasons underlying these negative effects are likely multifactorial involving different underlying pathophysiological mechanisms. Rapid and sizeable excess body weight loss can lead to nutritional deficiencies such as iron, calcium, and vitamins. This occurs in varying severity depending on the surgical procedure and can have a negative effect on spermatogenesis leading to worsening parameters (68). In addition, weight loss of such proportion can lead to release of liposoluble toxic substances that can cause alterations in spermatogenesis (68). A case series by Sermondade et al. analyzed three patients and found worsening sperm parameters in all three patients however in one case this effect was reversed after 24 months (68). This further supports the theory above suggesting that over time stabilization of nutritional status and reduction in toxic metabolites may have eventually led to improved sperm parameters (58). It is clear that there is no consensus currently in the scientific literature on the effects of bariatric surgery on sperm and semen parameters. Further studies with larger populations are required to elucidate exact effects and suggest underlying pathophysiological mechanisms.

## Conclusion

Obesity has become a global pandemic affecting both developed and developing nations. It has significant health consequences on the individual including issues relating to sexual and reproductive health. Bariatric surgery, as a metabolic treatment for obesity, is clearly very effective in terms of rapid and sustained reductions in BMI leading to restoration of reproductive and metabolic hormonal profile. Significant weight loss also alleviates the pro-inflammatory state that is associated with obesity and other co-morbid conditions and helps restore endothelial integrity thereby reducing erectile dysfunction and other cardiovascular risk burden. Bariatric surgery produces successful and encouraging outcomes in terms of the patient's overall health and well-being but more specifically seems to ameliorate hypogonadism and sexual dysfunction.

There remain several unanswered questions in relation to the effects of bariatric surgery on male fertility. The evidence of the effects of surgical interventions on sperm and semen parameters remains conflicting with no consensus reached in the current literature. It is clear from this review that most studies included are small and largely involve retrospective data analysis which is a clear area of improvement in future research and a limitation of this manuscript. Experimental medicine studies interrogating the elusive pathophysiology of obesity and male reproductive system can be invaluable in the optimisation of current and development of future tailored metabolic interventions to treat male obesity related reproductive dysfunction.

## Author Contributions

SS and GD wrote the manuscript. AP, SE-H, BW, RV, CR, FR, SA, and GD revised the final version of the manuscript. All authors contributed to the article and approved the submitted version.

## Conflict of Interest

The authors declare that the research was conducted in the absence of any commercial or financial relationships that could be construed as a potential conflict of interest. The handling Editor declared a past co-authorship with one of the authors GD.
